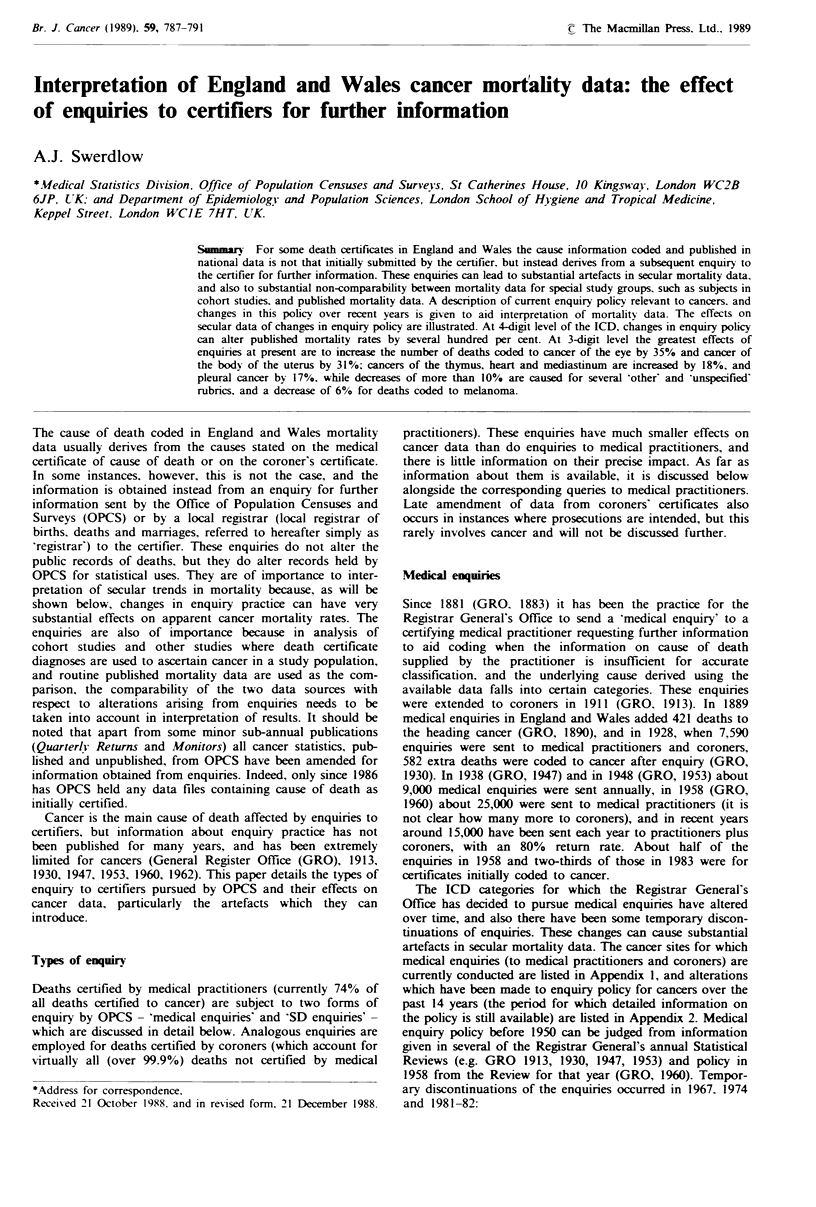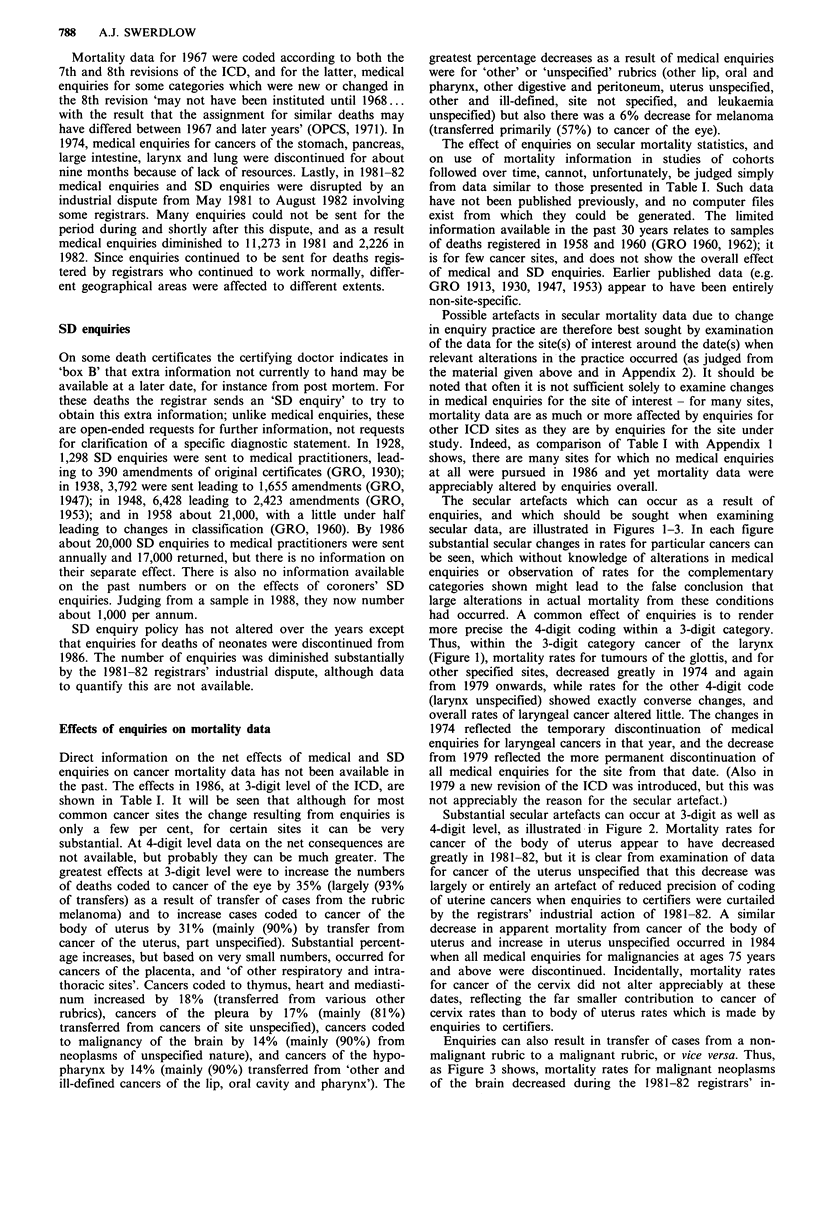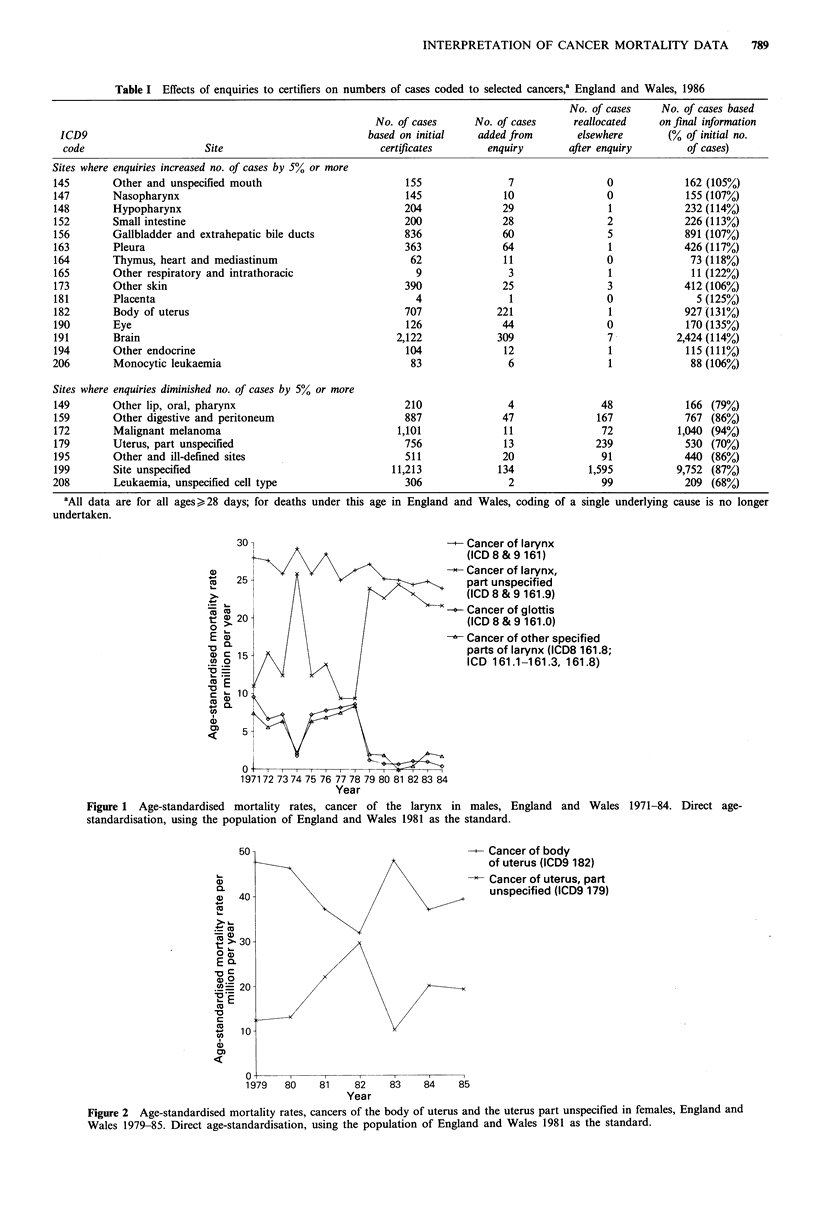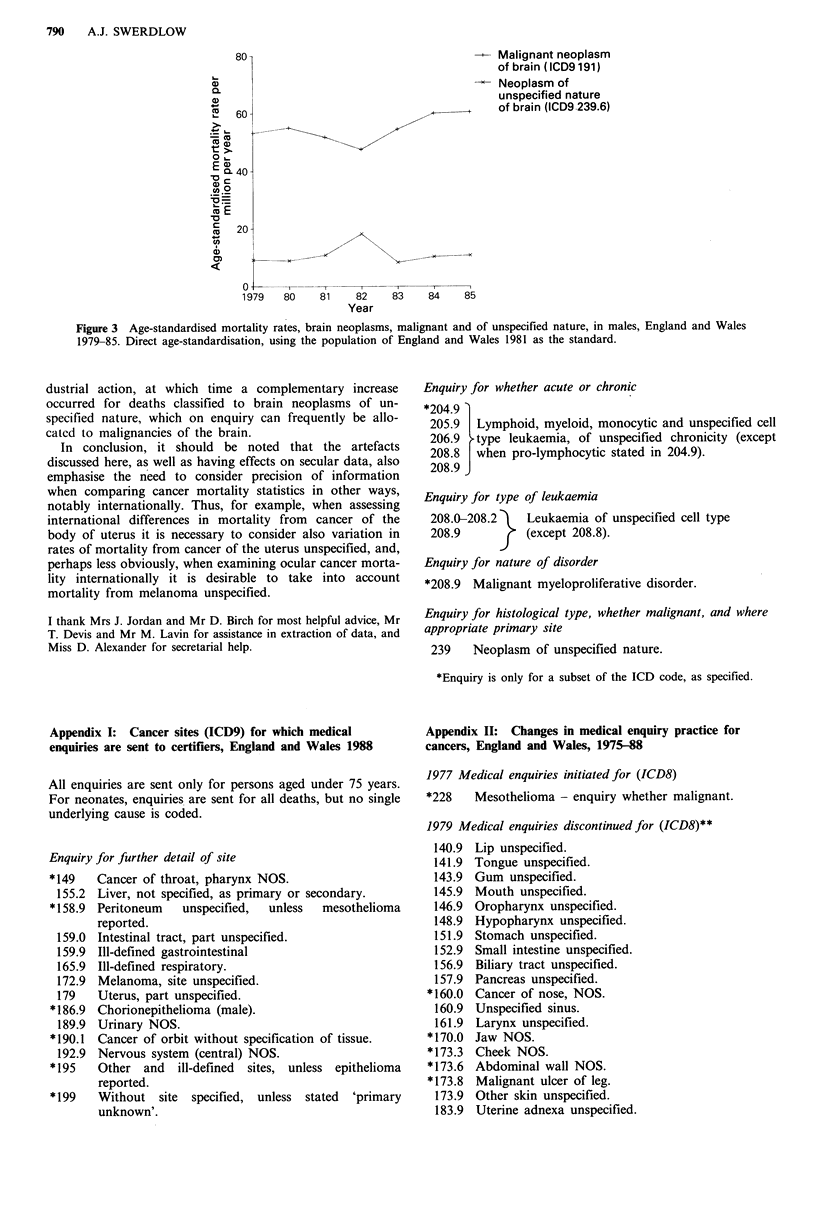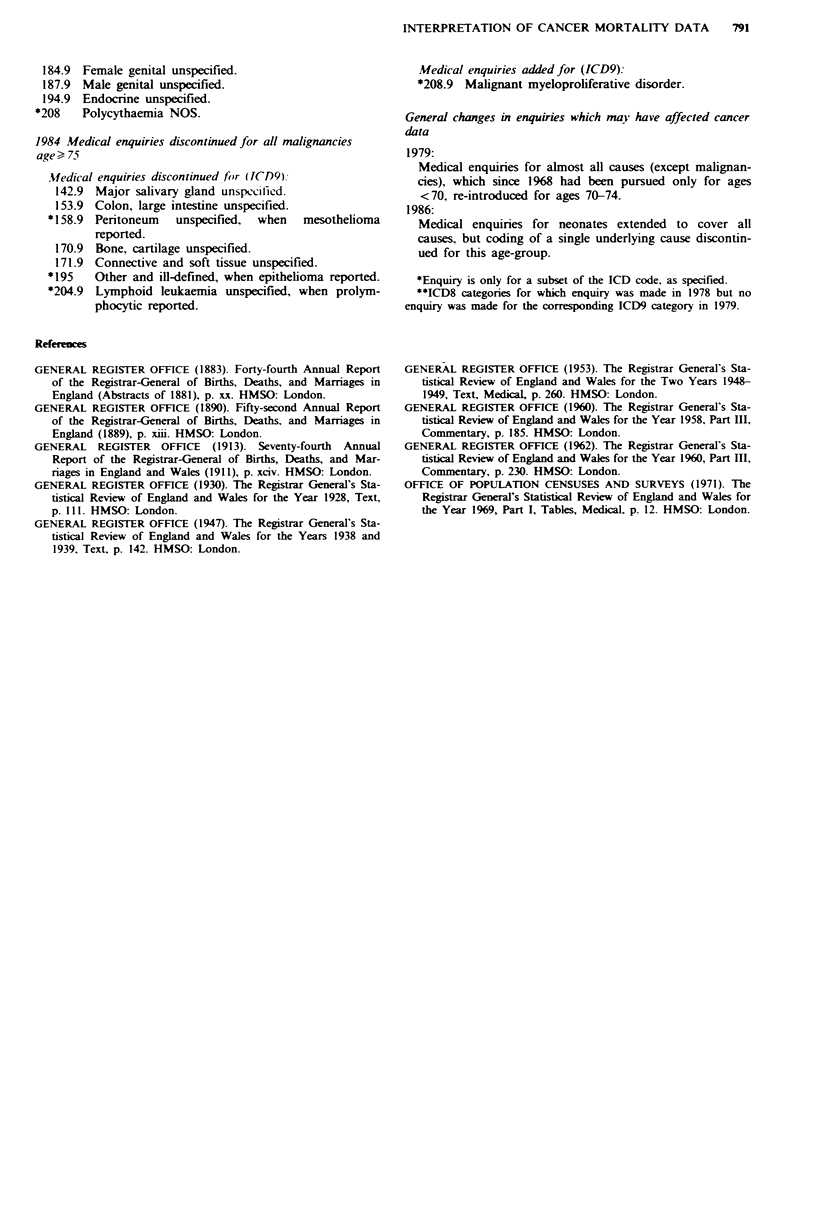# Interpretation of England and Wales cancer mortality data: the effect of enquiries to certifiers for further information.

**DOI:** 10.1038/bjc.1989.164

**Published:** 1989-05

**Authors:** A. J. Swerdlow

**Affiliations:** Medical Statistics Division, Office of Population Censuses and Surveys, London, UK.

## Abstract

For some death certificates in England and Wales the cause information coded and published in national data is not that initially submitted by the certifier, but instead derives from a subsequent enquiry to the certifier for further information. These enquiries can lead to substantial artefacts in secular mortality data, and also to substantial non-comparability between mortality data for special study groups, such as subjects in cohort studies, and published mortality data. A description of current enquiry policy relevant to cancers, and changes in this policy over recent years is given to aid interpretation of mortality data. The effects on secular data of changes in enquiry policy are illustrated. At 4-digit level of the ICD, changes in enquiry policy can alter published mortality rates by several hundred per cent. At 3-digit level the greatest effects of enquiries at present are to increase the number of deaths coded to cancer of the eye by 35% and cancer of the body of the uterus by 31%; cancers of the thymus, heart and mediastinum are increased by 18%, and pleural cancer by 17%, while decreases of more than 10% are caused for several 'other' and 'unspecified' rubrics, and a decrease of 6% for deaths coded to melanoma.


Br. J. Cancer (1989), 59, 787-791 © The Macmillan Press, Ltd., 1989

### Interpretation of England and Wales cancer mortality data: the effect of enquiries to certifiers for further information

A.J. Swerdlow

*Medical Statistics Division, Office of Population Censuses and Surveys, St Catherines House, 10 Kingsway, London WC2B 6JP, UK; and Department of Epidemiology and Population Sciences, London School of Hygiene and Tropical Medicine, Keppel Street, London WC1E 7HT, UK.

**Summary** For some death certificates in England and Wales the cause information coded and published in national data is not that initially submitted by the certifier, but instead derives from a subsequent enquiry to the certifier for further information. These enquiries can lead to substantial artefacts in secular mortality data, and also to substantial non-comparability between mortality data for special study groups, such as subjects in cohort studies, and published mortality data. A description of current enquiry policy relevant to cancers, and changes in this policy over recent years is given to aid interpretation of mortality data. The effects on secular data of changes in enquiry policy are illustrated. At 4-digit level of the ICD, changes in enquiry policy can alter published mortality rates by several hundred per cent. At 3-digit level the greatest effects of enquiries at present are to increase the number of deaths coded to cancer of the eye by 35% and cancer of the body of the uterus by 31%; cancers of the thymus, heart and mediastinum are increased by 18%, and pleural cancer by 17%, while decreases of more than 10% are caused for several 'other' and 'unspecified' rubrics, and a decrease of 6% for deaths coded to melanoma.

The cause of death coded in England and Wales mortality data usually derives from the causes stated on the medical certificate of cause of death or on the coroner's certificate. In some instances, however, this is not the case, and the information is obtained instead from an enquiry for further information sent by the Office of Population Censuses and Surveys (OPCS) or by a local registrar (local registrar of births, deaths and marriages, referred to hereafter simply as 'registrar') to the certifier. These enquiries do not alter the public records of deaths, but they do alter records held by OPCS for statistical uses. They are of importance to interpretation of secular trends in mortality because, as will be shown below, changes in enquiry practice can have very substantial effects on apparent cancer mortality rates. The enquiries are also of importance because in analysis of cohort studies and other studies where death certificate diagnoses are used to ascertain cancer in a study population, and routine published mortality data are used as the comparison, the comparability of the two data sources with respect to alterations arising from enquiries needs to be taken into account in interpretation of results. It should be noted that apart from some minor sub-annual publications (*Quarterly Returns* and *Monitors*) all cancer statistics, published and unpublished, from OPCS have been amended for information obtained from enquiries. Indeed, only since 1986 has OPCS held any data files containing cause of death as initially certified.

Cancer is the main cause of death affected by enquiries to certifiers, but information about enquiry practice has not been published for many years, and has been extremely limited for cancers (General Register Office (GRO), 1913, 1930, 1947, 1953, 1960, 1962). This paper details the types of enquiry to certifiers pursued by OPCS and their effects on cancer data, particularly the artefacts which they can introduce.

### Types of enquiry

Deaths certified by medical practitioners (currently 74% of all deaths certified to cancer) are subject to two forms of enquiry by OPCS – 'medical enquiries' and 'SD enquiries' – which are discussed in detail below. Analogous enquiries are employed for deaths certified by coroners (which account for virtually all (over 99.9%) deaths not certified by medical practitioners). These enquiries have much smaller effects on cancer data than do enquiries to medical practitioners, and there is little information on their precise impact. As far as information about them is available, it is discussed below alongside the corresponding queries to medical practitioners. Late amendment of data from coroners' certificates also occurs in instances where prosecutions are intended, but this rarely involves cancer and will not be discussed further.

### Medical enquiries

Since 1881 (GRO, 1883) it has been the practice for the Registrar General's Office to send a 'medical enquiry' to a certifying medical practitioner requesting further information to aid coding when the information on cause of death supplied by the practitioner is insufficient for accurate classification, and the underlying cause derived using the available data falls into certain categories. These enquiries were extended to coroners in 1911 (GRO, 1913). In 1889 medical enquiries in England and Wales added 421 deaths to the heading cancer (GRO, 1890), and in 1928, when 7,590 enquiries were sent to medical practitioners and coroners, 582 extra deaths were coded to cancer after enquiry (GRO, 1930). In 1938 (GRO, 1947) and in 1948 (GRO, 1953) about 9,000 medical enquiries were sent annually, in 1958 (GRO, 1960) about 25,000 were sent to medical practitioners (it is not clear how many more to coroners), and in recent years around 15,000 have been sent each year to practitioners plus coroners, with an 80% return rate. About half of the enquiries in 1958 and two-thirds of those in 1983 were for certificates initially coded to cancer.

The ICD categories for which the Registrar General's Office has decided to pursue medical enquiries have altered over time, and also there have been some temporary discontinuations of enquiries. These changes can cause substantial artefacts in secular mortality data. The cancer sites for which medical enquiries (to medical practitioners and coroners) are currently conducted are listed in Appendix 1, and alterations which have been made to enquiry policy for cancers over the past 14 years (the period for which detailed information on the policy is still available) are listed in Appendix 2. Medical enquiry policy before 1950 can be judged from information given in several of the Registrar General's annual Statistical Reviews (e.g. GRO 1913, 1930, 1947, 1953) and policy in 1958 from the Review for that year (GRO, 1960). Temporary discontinuations of the enquiries occurred in 1967, 1974 and 1981–82:

*Address for correspondence.

Received 21 October 1988, and in revised form, 21 December 1988.

Mortality data for 1967 were coded according to both the 7th and 8th revisions of the ICD, and for the latter, medical enquiries for some categories which were new or changed in the 8th revision 'may not have been instituted until 1968... with the result that the assignment for similar deaths may have differed between 1967 and later years' (OPCS, 1971). In 1974, medical enquiries for cancers of the stomach, pancreas, large intestine, larynx and lung were discontinued for about nine months because of lack of resources. Lastly, in 1981–82 medical enquiries and SD enquiries were disrupted by an industrial dispute from May 1981 to August 1982 involving some registrars. Many enquiries could not be sent for the period during and shortly after this dispute, and as a result medical enquiries diminished to 11,273 in 1981 and 2,226 in 1982. Since enquiries continued to be sent for deaths registered by registrars who continued to work normally, different geographical areas were affected to different extents.

### SD enquiries

On some death certificates the certifying doctor indicates in 'box B' that extra information not currently to hand may be available at a later date, for instance from post mortem. For these deaths the registrar sends an 'SD enquiry' to try to obtain this extra information; unlike medical enquiries, these are open-ended requests for further information, not requests for clarification of a specific diagnostic statement. In 1928, 1,298 SD enquiries were sent to medical practitioners, leading to 390 amendments of original certificates (GRO, 1930); in 1938, 3,792 were sent leading to 1,655 amendments (GRO, 1947); in 1948, 6,428 leading to 2,423 amendments (GRO, 1953); and in 1958 about 21,000, with a little under half leading to changes in classification (GRO, 1960). By 1986 about 20,000 SD enquiries to medical practitioners were sent annually and 17,000 returned, but there is no information on their separate effect. There is also no information available on the past numbers or on the effects of coroners' SD enquiries. Judging from a sample in 1988, they now number about 1,000 per annum.

SD enquiry policy has not altered over the years except that enquiries for deaths of neonates were discontinued from 1986. The number of enquiries was diminished substantially by the 1981–82 registrars' industrial dispute, although data to quantify this are not available.

### Effects of enquiries on mortality data

Direct information on the net effects of medical and SD enquiries on cancer mortality data has not been available in the past. The effects in 1986, at 3-digit level of the ICD, are shown in Table I. It will be seen that although for most common cancer sites the change resulting from enquiries is only a few per cent, for certain sites it can be very substantial. At 4-digit level data on the net consequences are not available, but probably they can be much greater. The greatest effects at 3-digit level were to increase the numbers of deaths coded to cancer of the eye by 35% (largely (93% of transfers) as a result of transfer of cases from the rubric melanoma) and to increase cases coded to cancer of the body of uterus by 31% (mainly (90%) by transfer from cancer of the uterus, part unspecified). Substantial percentage increases, but based on very small numbers, occurred for cancers of the placenta, and 'of other respiratory and intrathoracic sites'. Cancers coded to thymus, heart and mediastinum increased by 18% (transferred from various other rubrics), cancers of the pleura by 17% (mainly (81%) transferred from cancers of site unspecified), cancers coded to malignancy of the brain by 14% (mainly (90%) from neoplasms of unspecified nature), and cancers of the hypopharynx by 14% (mainly (90%) transferred from 'other and ill-defined cancers of the lip, oral cavity and pharynx'). The greatest percentage decreases as a result of medical enquiries were for 'other' or 'unspecified' rubrics (other lip, oral and pharynx, other digestive and peritoneum, uterus unspecified, other and ill-defined, site not specified, and leukaemia unspecified) but also there was a 6% decrease for melanoma (transferred primarily (57%) to cancer of the eye).

The effect of enquiries on secular mortality statistics, and on use of mortality information in studies of cohorts followed over time, cannot, unfortunately, be judged simply from data similar to those presented in Table I. Such data have not been published previously, and no computer files exist from which they could be generated. The limited information available in the past 30 years relates to samples of deaths registered in 1958 and 1960 (GRO 1960, 1962); it is for few cancer sites, and does not show the overall effect of medical and SD enquiries. Earlier published data (e.g. GRO 1913, 1930, 1947, 1953) appear to have been entirely non-site-specific.

Possible artefacts in secular mortality data due to change in enquiry practice are therefore best sought by examination of the data for the site(s) of interest around the date(s) when relevant alterations in the practice occurred (as judged from the material given above and in Appendix 2). It should be noted that often it is not sufficient solely to examine changes in medical enquiries for the site of interest – for many sites, mortality data are as much or more affected by enquiries for other ICD sites as they are by enquiries for the site under study. Indeed, as comparison of Table I with Appendix 1 shows, there are many sites for which no medical enquiries at all were pursued in 1986 and yet mortality data were appreciably altered by enquiries overall.

The secular artefacts which can occur as a result of enquiries, and which should be sought when examining secular data, are illustrated in Figures 1–3. In each figure substantial secular changes in rates for particular cancers can be seen, which without knowledge of alterations in medical enquiries or observation of rates for the complementary categories shown might lead to the false conclusion that large alterations in actual mortality from these conditions had occurred. A common effect of enquiries is to render more precise the 4-digit coding within a 3-digit category. Thus, within the 3-digit category cancer of the larynx (Figure 1), mortality rates for tumours of the glottis, and for other specified sites, decreased greatly in 1974 and again from 1979 onwards, while rates for the other 4-digit code (larynx unspecified) showed exactly converse changes, and overall rates of laryngeal cancer altered little. The changes in 1974 reflected the temporary discontinuation of medical enquiries for laryngeal cancers in that year, and the decrease from 1979 reflected the more permanent discontinuation of all medical enquiries for the site from that date. (Also in 1979 a new revision of the ICD was introduced, but this was not appreciably the reason for the secular artefact.)

Substantial secular artefacts can occur at 3-digit as well as 4-digit level, as illustrated in Figure 2. Mortality rates for cancer of the body of uterus appear to have decreased greatly in 1981–82, but it is clear from examination of data for cancer of the uterus unspecified that this decrease was largely or entirely an artefact of reduced precision of coding of uterine cancers when enquiries to certifiers were curtailed by the registrars' industrial action of 1981–82. A similar decrease in apparent mortality from cancer of the body of uterus and increase in uterus unspecified occurred in 1984 when all medical enquiries for malignancies at ages 75 years and above were discontinued. Incidentally, mortality rates for cancer of the cervix did not alter appreciably at these dates, reflecting the far smaller contribution to cancer of cervix rates than to body of uterus rates which is made by enquiries to certifiers.

Enquiries can also result in transfer of cases from a non-malignant rubric to a malignant rubric, or *vice versa*. Thus, as Figure 3 shows, mortality rates for malignant neoplasms of the brain decreased during the 1981–82 registrars' in-

**Table I** Effects of enquiries to certifiers on numbers of cases coded to selected cancers,[a] England and Wales, 1986

| *ICD9 code* | *Site* | *No. of cases based on initial certificates* | *No. of cases added from enquiry* | *No. of cases reallocated elsewhere after enquiry* | *No. of cases based on final information (% of initial no. of cases)* |
|---|---|---|---|---|---|
| *Sites where enquiries increased no. of cases by 5% or more* | | | | | |
| 145 | Other and unspecified mouth | 155 | 7 | 0 | 162 (105%) |
| 147 | Nasopharynx | 145 | 10 | 0 | 155 (107%) |
| 148 | Hypopharynx | 204 | 29 | 1 | 232 (114%) |
| 152 | Small intestine | 200 | 28 | 2 | 226 (113%) |
| 156 | Gallbladder and extrahepatic bile ducts | 836 | 60 | 5 | 891 (107%) |
| 163 | Pleura | 363 | 64 | 1 | 426 (117%) |
| 164 | Thymus, heart and mediastinum | 62 | 11 | 0 | 73 (118%) |
| 165 | Other respiratory and intrathoracic | 9 | 3 | 1 | 11 (122%) |
| 173 | Other skin | 390 | 25 | 3 | 412 (106%) |
| 181 | Placenta | 4 | 1 | 0 | 5 (125%) |
| 182 | Body of uterus | 707 | 221 | 1 | 927 (131%) |
| 190 | Eye | 126 | 44 | 0 | 170 (135%) |
| 191 | Brain | 2,122 | 309 | 7 | 2,424 (114%) |
| 194 | Other endocrine | 104 | 12 | 1 | 115 (111%) |
| 206 | Monocytic leukaemia | 83 | 6 | 1 | 88 (106%) |
| *Sites where enquiries diminished no. of cases by 5% or more* | | | | | |
| 149 | Other lip, oral, pharynx | 210 | 4 | 48 | 166 (79%) |
| 159 | Other digestive and peritoneum | 887 | 47 | 167 | 767 (86%) |
| 172 | Malignant melanoma | 1,101 | 11 | 72 | 1,040 (94%) |
| 179 | Uterus, part unspecified | 756 | 13 | 239 | 530 (70%) |
| 195 | Other and ill-defined sites | 511 | 20 | 91 | 440 (86%) |
| 199 | Site unspecified | 11,213 | 134 | 1,595 | 9,752 (87%) |
| 208 | Leukaemia, unspecified cell type | 306 | 2 | 99 | 209 (68%) |

[a]All data are for all ages≥28 days; for deaths under this age in England and Wales, coding of a single underlying cause is no longer undertaken.

**Figure 1** Age-standardised mortality rates, cancer of the larynx in males, England and Wales 1971–84. Direct age-standardisation, using the population of England and Wales 1981 as the standard.

**Figure 2** Age-standardised mortality rates, cancers of the body of uterus and the uterus part unspecified in females, England and Wales 1979–85. Direct age-standardisation, using the population of England and Wales 1981 as the standard.

**Figure 3** Age-standardised mortality rates, brain neoplasms, malignant and of unspecified nature, in males, England and Wales 1979–85. Direct age-standardisation, using the population of England and Wales 1981 as the standard.

dustrial action, at which time a complementary increase occurred for deaths classified to brain neoplasms of unspecified nature, which on enquiry can frequently be allocated to malignancies of the brain.

In conclusion, it should be noted that the artefacts discussed here, as well as having effects on secular data, also emphasise the need to consider precision of information when comparing cancer mortality statistics in other ways, notably internationally. Thus, for example, when assessing international differences in mortality from cancer of the body of uterus it is necessary to consider also variation in rates of mortality from cancer of the uterus unspecified, and, perhaps less obviously, when examining ocular cancer mortality internationally it is desirable to take into account mortality from melanoma unspecified.

I thank Mrs J. Jordan and Mr D. Birch for most helpful advice, Mr T. Devis and Mr M. Lavin for assistance in extraction of data, and Miss D. Alexander for secretarial help.

### Appendix I: Cancer sites (ICD9) for which medical enquiries are sent to certifiers, England and Wales 1988

All enquiries are sent only for persons aged under 75 years. For neonates, enquiries are sent for all deaths, but no single underlying cause is coded.

*Enquiry for further detail of site*

*149 Cancer of throat, pharynx NOS.
155.2 Liver, not specified, as primary or secondary.
*158.9 Peritoneum unspecified, unless mesothelioma reported.
159.0 Intestinal tract, part unspecified.
159.9 Ill-defined gastrointestinal
165.9 Ill-defined respiratory.
172.9 Melanoma, site unspecified.
179 Uterus, part unspecified.
*186.9 Chorionepithelioma (male).
189.9 Urinary NOS.
*190.1 Cancer of orbit without specification of tissue.
192.9 Nervous system (central) NOS.
*195 Other and ill-defined sites, unless epithelioma reported.
*199 Without site specified, unless stated 'primary unknown'.

*Enquiry for whether acute or chronic*

*204.9, 205.9, 206.9, 208.8, 208.9 — Lymphoid, myeloid, monocytic and unspecified cell type leukaemia, of unspecified chronicity (except when pro-lymphocytic stated in 204.9).

*Enquiry for type of leukaemia*

208.0–208.2, 208.9 — Leukaemia of unspecified cell type (except 208.8).

*Enquiry for nature of disorder*

*208.9 Malignant myeloproliferative disorder.

*Enquiry for histological type, whether malignant, and where appropriate primary site*

239 Neoplasm of unspecified nature.

*Enquiry is only for a subset of the ICD code, as specified.

### Appendix II: Changes in medical enquiry practice for cancers, England and Wales, 1975–88

*1977 Medical enquiries initiated for (ICD8)*

*228 Mesothelioma – enquiry whether malignant.

*1979 Medical enquiries discontinued for (ICD8)***

140.9 Lip unspecified.

141.9 Tongue unspecified.

143.9 Gum unspecified.

145.9 Mouth unspecified.

146.9 Oropharynx unspecified.

148.9 Hypopharynx unspecified.

151.9 Stomach unspecified.

152.9 Small intestine unspecified.

156.9 Biliary tract unspecified.

157.9 Pancreas unspecified.

*160.0 Cancer of nose, NOS.

160.9 Unspecified sinus.

161.9 Larynx unspecified.

*170.0 Jaw NOS.

*173.3 Cheek NOS.

*173.6 Abdominal wall NOS.

*173.8 Malignant ulcer of leg.

173.9 Other skin unspecified.

183.9 Uterine adnexa unspecified.

184.9 Female genital unspecified.
187.9 Male genital unspecified.
194.9 Endocrine unspecified.
*208 Polycythaemia NOS.

*1984 Medical enquiries discontinued for all malignancies age ≥ 75*

*Medical enquiries discontinued for (ICD9):*
142.9 Major salivary gland unspecified.
153.9 Colon, large intestine unspecified.
*158.9 Peritoneum unspecified, when mesothelioma reported.
170.9 Bone, cartilage unspecified.
171.9 Connective and soft tissue unspecified.
*195 Other and ill-defined, when epithelioma reported.
*204.9 Lymphoid leukaemia unspecified, when prolymphocytic reported.

*Medical enquiries added for (ICD9):*
*208.9 Malignant myeloproliferative disorder.

*General changes in enquiries which may have affected cancer data*

1979:
Medical enquiries for almost all causes (except malignancies), which since 1968 had been pursued only for ages <70, re-introduced for ages 70–74.

1986:
Medical enquiries for neonates extended to cover all causes, but coding of a single underlying cause discontinued for this age-group.

*Enquiry is only for a subset of the ICD code, as specified.
**ICD8 categories for which enquiry was made in 1978 but no enquiry was made for the corresponding ICD9 category in 1979.

**References**

GENERAL REGISTER OFFICE (1883). Forty-fourth Annual Report of the Registrar-General of Births, Deaths, and Marriages in England (Abstracts of 1881), p. xx. HMSO: London.
GENERAL REGISTER OFFICE (1890). Fifty-second Annual Report of the Registrar-General of Births, Deaths, and Marriages in England (1889), p. xiii. HMSO: London.
GENERAL REGISTER OFFICE (1913). Seventy-fourth Annual Report of the Registrar-General of Births, Deaths, and Marriages in England and Wales (1911), p. xciv. HMSO: London.
GENERAL REGISTER OFFICE (1930). The Registrar General's Statistical Review of England and Wales for the Year 1928, Text, p. 111. HMSO: London.
GENERAL REGISTER OFFICE (1947). The Registrar General's Statistical Review of England and Wales for the Years 1938 and 1939, Text, p. 142. HMSO: London.
GENERAL REGISTER OFFICE (1953). The Registrar General's Statistical Review of England and Wales for the Two Years 1948–1949, Text, Medical, p. 260. HMSO: London.
GENERAL REGISTER OFFICE (1960). The Registrar General's Statistical Review of England and Wales for the Year 1958, Part III, Commentary, p. 185. HMSO: London.
GENERAL REGISTER OFFICE (1962). The Registrar General's Statistical Review of England and Wales for the Year 1960, Part III, Commentary, p. 230. HMSO: London.
OFFICE OF POPULATION CENSUSES AND SURVEYS (1971). The Registrar General's Statistical Review of England and Wales for the Year 1969, Part I, Tables, Medical, p. 12. HMSO: London.